# Novel Antiviral Efficacy of *Hedyotis diffusa* and *Artemisia capillaris* Extracts against Dengue Virus, Japanese Encephalitis Virus, and Zika Virus Infection and Immunoregulatory Cytokine Signatures

**DOI:** 10.3390/plants11192589

**Published:** 2022-09-30

**Authors:** Zhan Qiu Mao, Noboru Minakawa, Meng Ling Moi

**Affiliations:** 1 Institute of Tropical Medicine, Graduate School of Biomedical Sciences, Nagasaki University, Nagasaki 852-8523, Japan; 2 School of International Health, Graduate School of Medicine, The University of Tokyo, Tokyo 113-0033, Japan

**Keywords:** *Hedyotis diffusa*, *Artemisia capillaris*, inhibition, flavivirus, Japanese encephalitis virus (JEV), Zika virus (ZIKV), dengue virus (DENV), inflammatory cytokines

## Abstract

Currently, there are no specific therapeutics for flavivirus infections, including dengue virus (DENV) and Zika virus (ZIKV). In this study, we evaluated extracts from the plants *Hedyotis diffusa* (HD) and *Artemisia capillaris* (AC) to determine the antiviral activity against DENV, ZIKV, and Japanese encephalitis virus (JEV). HD and AC demonstrated inhibitory activity against JEV, ZIKV, and DENV replication and reduced viral RNA levels in a dose–responsive manner, with non-cytotoxic concentration ranging from 0.1 to 10 mg/mL. HD and AC had low cytotoxicity to Vero cells, with CC_50_ values of 33.7 ± 1.6 and 30.3 ± 1.7 mg/mL (mean ± SD), respectively. The anti-flavivirus activity of HD and AC was also consistent in human cell lines, including human glioblastoma (T98G), human chronic myeloid leukemia (K562), and human embryonic kidney (HEK-293T) cells. Viral-infected, HD-treated cells demonstrated downregulation of cytokines including CCR1, CCL26, CCL15, CCL5, IL21, and IL17C. In contrast, CCR1, CCL26, and AIMP1 were elevated following AC treatment in viral-infected cells. Overall, HD and AC plant extracts demonstrated flavivirus replication inhibitory activity, and together with immunoregulatory cytokine signatures, these results suggest that HD and AC possess bioactive compounds that may further be refined as promising candidates for clinical applications.

## 1. Introduction

Over a third of the global population are at risk of flavivirus infection [1,2,3]. In the recent decade, flaviviruses have caused several global epidemics with high morbidity and mortality rates, in which a majority of these epidemics were caused by dengue virus (DENV), Zika virus (ZIKV), and Japanese encephalitis virus (JEV) [4,5]. Flaviviruses are encoded by a positive-sense single-stranded RNA that can immediately hijack the ribosome for translation [4,6]. The vast majority of flavivirus infections involve an intermediate host prior to transmission to humans through the biting by arthropods, such as mosquitoes in the case of DENV, ZIKV, and JEV [4,7].

Dengue is the most prevalent mosquito-borne, extensively spread, and highly endemic viral infectious disease throughout tropical and sub-tropical regions [8,9]. There are four antigenically distinct serotypes of DENV, designated as DENV-1, DENV-2, DENV-3, and DENV-4; all cause a spectrum of symptoms [10,11]. The most severe clinical syndrome can manifest in the form of dengue shock syndrome (DSS), with the most prominent clinical presentation being plasma leakage with hypovolemic shock and multi-organ failure [12,13,14]. Dengue is endemic in more than 100 countries and the incidence of dengue has significantly increased around the world in recent decades, in which 390 million dengue infections per year have been estimated to occur globally [15,16,17]. Infection with any of the four serotypes probably induces life-long immunity, but this does not confer protective immunity to the other three serotypes [18,19]. Instead, a second heterotypic infection can result in an antibody-dependent enhancement (ADE) effect which accounts for vascular endothelial cell dysfunction and increased vascular permeability [20,21,22]. The limited understanding on ADE in dengue disease protection has hampered the development of a universal vaccine for all dengue serotypes [4,23]. ZIKV, a closely related flavivirus to dengue, was first isolated from the serum of a sentinel rhesus macaque monkey in Uganda in 1947 [24]. The antibody against ZIKV in human serum was first detected in 1952 [25,26]. This virus is transmitted by daytime-active *Aedes* mosquitoes, is circulating in the Pacific regions, Americas, Southeast Asia, and West Africa, and has infected an estimated 1.62 million people in more than 70 countries globally [27,28]. ZIKV remains an important cause of congenital infection, fetal microcephaly, and Guillain-Barré syndrome in populations [29]. As of 2015, the ZIKV pandemic in the Americas was associated with several thousand newborns with microcephaly [25,30,31]. Japanese encephalitis (JE) remains one of the major flavivirus threats in Asia and the Western Pacific region. JE is an acute infectious disease of the central nervous system (CNS) caused by JEV [32]. It circulates among the mosquitoes of the genus Culex and vertebrate hosts, particularly pigs and wild wading birds, and humans are incidental or dead-end hosts [33,34,35]. Although JEV vaccines are used extensively in most endemic countries, the disease eradication is challenging due to its zoonotic life cycle [36]. The case fatality rate of symptomatic cases is up to 30% among the symptomatic cases of JEV. Of those who survive, 30% to 50% of the surviving patients had long-term neurological, psychological, and cognitive impairment, including polio-like weakness and life-long seizure disorders [37,38].

In this context, there is an urgent need for effective therapeutic interventions against flaviviruses [39,40,41,42]. *Hedyotis diffusa* (HD) and *Artemisia capillaris* (AC) are annual herbs used for Wen Bing (“warm disease”) treatment in traditional Chinese medicine (TCM) [43]. TCM describes HD and AC as herbs that possess capabilities in clearing away the “evil heat” (i.e., fevers, including those that are a result of infection). Additionally, TCM views HD and AC herbs as approaches in “dispelling evil”, which may correspond to either pathogen inhibition, immune regulation, inflammation control, or host tissue protection [44,45,46]. As TCM does not identify the specific therapeutic target and mechanisms in the context of contemporary biological theories, there are limited data on the efficacy and mechanisms of HD and AC against virus infections. In line with this, the present study aims to determine the anti-flaviviral activity of HD and AC extracts against (1) DENV, (2) ZIKV, and (3) JEV with dose–response assessment and time-of-drug assay by using human and mammalian cell lines.

## 2. Materials and Methods

### 2.1. Herbs

The concentrated Chinese medicine granules (CCMG) of *Hedyotis diffusa* (HD) and *Artemisia capillaris* (AC) (Guangdong Yifang Pharmaceutical Co., Ltd., Foshan, China) are crude aqueous extracts from 15 g raw herb plants. Both CCMGs were dissolved in 15 mL of EMEM supplemented with heat-inactivated 10% FCS, then passed through a sterile 0.45 µm membrane filtration (Millex-HV filter, Merck Millipore Ltd., Tullagreen, Ireland) to make stock solutions, which equated to 1000 mg raw herbs per mL (1000 mg/mL). The concentrate was further aliquoted into smaller sizes and stored at -20°C until further experiments. All stored herbs were used once after thaw.

### 2.2. Cell Lines

African green monkey kidney (Vero 9013) and baby hamster kidney (BHK-21) cell lines were maintained in Eagle’s minimum essential medium (EMEM) supplemented with 10% fetal calf serum (FCS). Human glioblastoma (T98G) cell lines were maintained in Roswell Park Memorial Institute 1640 (RPMI, Gibco, Grand Island, NY, USA) Medium supplemented with 15% FCS and 100 units/mL penicillin. Human chronic myeloid leukemia (K562) cell lines were grown in RPMI containing 10% FCS and 100 units/mL penicillin. Human embryonic kidney (HEK-293T) cell lines were cultured in Dulbecco’s Modified Eagle Medium (DMEM, Gibco, Paisley, UK), supplemented with 10% FCS and 100 units/mL penicillin. All the FCS was heat-inactivated without antibiotics, and all cell lines were cultured at 37 °C in a humidified atmosphere of 5% CO_2_.

### 2.3. Viruses

Three virus strains were used in this study: (1) JEV OH0566 strain (GenBank accession no. AY508813); (2) ZIKV PRVABC59 strain (GenBank accession no. KU501215.1); and (3) DENV-2 TL-30 strain (GenBank accession no. AB219135). JEV OH0566 and ZIKV PRVABC59 were propagated in Vero 9013 cells, and DENV-2 TL-30 was propagated in BHK-21, at 37 °C in a humidified atmosphere of 5% CO_2_ for up to 5 days [10]. Infected culture fluids (ICF) were collected, clarified by centrifugation at 3,000 RPM for 10 min at 4 °C, and stored in aliquots at −80 °C until use. Virus titers (plaque-forming units (PFU) per mL) were determined by plaque assay, and the number of genome copies was determined by quantitative RT-qPCR.

### 2.4. Cell Cytotoxicity Assay and CC_50_ Determination

The cytotoxicity of herbs was determined by using MTT cell proliferation assay kit (Cayman Chemical, Michigan, MI, USA), following the manufacturer’s protocol. Briefly, cells were seeded in a 96-well plate at a density of 2 × 10^4^ cells/well. After 24 h, cells were overlaid with media containing various concentrations (0.1–100 mg/mL) of herb or EMEM supplemented with 10% FCS and were cultured for an additional four days at 37 °C in a humidified atmosphere of 5% CO_2_. A total of 10 µL of MTT reagent was added into the cell culture supernatant. The MTT-reagent-treated cells were cultured at 37 °C in a humidified atmosphere of 5% CO_2_. Four hours later, 100 µL of crystal dissolving solution was added into the cell culture supernatant and the mixture was incubated for 18 h at 37 °C in a humidified atmosphere of 5% CO_2_. The absorbance of each sample was measured at 570 nm using a Synergy microplate reader (BioTek). Cytotoxicity was determined with the following equation using absorbance: cell viability (%) = [ OD value of treated cells − mean OD of wells without cells]/[ mean OD value of untreated cells − mean OD of wells without cells] ×100% (OD represents optical density). Half maximal cytotoxic concentration (CC_50_) value was calculated using GraphPad Prism. Each assay was conducted in double replicates and three experimental repeats.

### 2.5. Virus Infection

Vero 9013 cells (2 × 10^4^ cells/well) were cultured in a 96-well plate at 37 °C in a humidified atmosphere of 5% CO_2_ for 24h. Subsequently, the cells were infected with a ten-fold serial dilution of virus stock at multiplicity of infection (MOI) values of 0.1, 0.01, and 0.001, respectively. After 1 h incubation, cells were washed twice with EMEM and supplemented with 200 µL fresh EMEM/10% FCS and cultured at 37 °C in a humidified atmosphere of 5% CO_2_. At 24, 48, 72, and 96 h post infection (h p.i.), the cell culture supernatants were harvested, and viral titer (PFU) was determined via plaque assay.

### 2.6. Plaque Assay

The plaque assay was performed to determine the levels of infectious viral particles. Viral supernatant from infected cells was harvested on day 3 (72 h p.i.) after inoculation for JEV OH0566 and ZIKV PRVABC59 and day 4 (96 h p.i.) after inoculation for DENV-2 TL-30. Briefly, 25 μL of virus-infected cell culture was added into 225µL EMEM, followed by a ten-fold serial dilution in EMEM. A total of 100 µL of serial diluted liquid was inoculated onto monolayers of Vero cells in a 12-well plate. Next, the plates were incubated at 37 °C in a humidified atmosphere of 5% CO_2_ up to 60 min, and after incubation, 2 mL of overlay media (5 g of Methylcellulose (Wako Pure Chemical Industries Ltd., Osaka, Japan), 12 g of Avicel RC 591 (FMC Biopolymer, Philadelphia, PE, USA), and 9.4 g of EMEM powder was dissolved in 1 L of DDW) was added to each well. After (1) 4 days p.i. for JEV OH0566 and ZIKV PRVABC59, and (2) 5 days p.i. for DENV-2 TL-30, cells were fixed with 4% paraformaldehyde and stained with 0.25% crystal violet (Wako Pure Chemical Industries) [47]. The number of plaques were counted with the naked eye and viral titer was defined as plaque-forming units per milliliter (PFU/mL).

### 2.7. Antiviral Assay and EC_50_ Determination

Vero 9013 cells were seeded in 96-well plates (2 × 10^4^ cells/well) and incubated at 37 °C in a humidified atmosphere of 5% CO_2_. After 24 h, the cells were infected with each virus at multiplicity of infection (MOI) of 0.01 and incubated for 1 h. The cells were washed with EMEM and cultured in a culture medium containing various concentrations (0.1-10 mg/mL) of herb. After 3 days (JEV OH0566 and ZIKV PRVABC59) and 4 days (DENV-2 TL-30) of incubation, the cell culture supernatants were harvested for viral progeny determination via plaque assay and quantitative detection of viral RNA copy using real-time RT-qPCR. Inhibition (%) was determined by using the following equation: Inhibition (%) = (viral titer (log_10_ PFU/mL) of treated culture supernatants/mean viral titer (log_10_ PFU/mL) of untreated viral-infected control culture supernatants) ×100%. Half maximal effective concentration (EC_50_) value was calculated using GraphPad Prism. Selectivity index (SI) for herbs was determined as the ratio of CC_50_:EC_50_. Each assay was conducted in double replicates and three independent experiments.

### 2.8. Viral RNA Extraction

Viral RNA was extracted from 100 µL of virus-infected cell cultures using Quick Viral RNA kit (Zymo research, Irvine, CA, USA), following the manufacturer’s protocol. The eluted RNA samples were either used immediately or stored at −80 °C pending analysis.

### 2.9. Real-Time Quantitative Reverse-Transcription Polymerase Chain Reaction

A range of ten-fold serial dilution of in vitro transcribed RNA from 10^7^ to 10^3^ was used to generate the standard curve [48]. Gene-specific primers and probes targeting the envelope protein for DENV-2 TL-30 [48], ZIKV PRVABC59 [49], and JEV OH0566 [50] were used (Supplementary Table S1). The viral RNA levels were defined as log10 viral genome copies per mL. PCR master mix consisted of 5 µL of RNA, 5 µL of TaqMan Fast Virus 1-Step Master Mix (Applied Biosystems, Waltham, MA, USA), 0.25 µL of 100 µM forward and reverse primer, 0.5 µL of 10 µM probe, and 9 µL of nuclease-free water. The experiment and the following real-time PCR program on ABI instrument was set up as follows: 50 °C 5 min, 1 cycle; 95 °C 20 s, 1 cycle; 95 °C 3 s, 60 °C 30 s, 40 cycles. The mixture was added to the reaction to detect the amplification of target viral RNA (QuantStudio 7 Flex, Thermo Fisher Scientific, Waltham, MA, USA). The real-time RT-qPCR results were analyzed with the QuantStudio™ Real-Time PCR Software ver. 1.1 and the amplification plots were reviewed for baseline and threshold value correction. All isolated RNA and synthetic RNA were stored in −80 °C.

### 2.10. Time of Drug Addition Assay

Vero 9013 cells at a density of 2 × 10^4^ cells per well were seeded on 96-well plates and incubated for 24 h at 37 °C in a humidified atmosphere of 5% CO_2_. Cells were infected with each virus at MOI 0.01 and treated with each herb (5 mg/mL) at (1) one hour prior to infection, (2) simultaneously, or (3) one hour post infection, respectively. (1) Pre-treatment (time = −1 h p.i.): Vero 9013 cells were treated with herbs at a concentration of 5 mg/mL at 1 h prior to virus (MOI = 0.01) infection; (2) Co-treatment (time = 0 h p.i.): Vero 9013 were treated with herbs at 5 mg/mL, and infected with virus (MOI = 0.01) at the same time; (3) post-treatment (time = 1 h p.i.): Vero 9013 cells were infected with virus (MOI = 0.01) for 1 h, viral inoculum was aspirated, cells were washed with EMEM twice, and replaced with fresh medium containing each herb (5 mg/mL). After an additional 3 days for ZIKV PRVABC59 and JEV OH0566, or 4 days for DENV-2 TL-30, the cell culture supernatants were harvested for viral progeny determination via plaque assay. The time of incubation for each virus was determined as the optimal time for the detection of virus in the supernatant (data not shown). The pre- and co-treated cells were not washed, and there remains a possibility of unbound virus in the supernatant; as such, the virus mixture in the corresponding untreated viral-infected control supernatant was not removed, for synchronizing the infection in the same condition. However, the unbound virus in the post-treated group was removed; accordingly, the corresponding untreated viral-infected control cells were washed by fresh medium to remove the unbound virus.

### 2.11. RT^2^ Profiler PCR Arrays

Total RNA from T98G cells was isolated using Qiagen RNeasy Mini kit (Qiagen, Hilden, Germany) according to the manufacturer’s instructions. The concentration of eluted RNA samples was measured using Qubit® RNA BR Assay Kits (Invitrogen, Thermo Fisher Scientific, Waltham, MA, USA). cDNA synthesis was carried out using 3.0 μg total RNA sample as a template with the RT^2^ First Strand Kit (Qiagen, Maryland, MD, USA). Human Inflammatory Cytokines and Receptors RT^2^ Profiler PCR Arrays (PAHS-011ZC-12; Qiagen, Maryland, USA) were used in the present study. A 96-well plate which contained 84 target genes, a housekeeping gene panel to normalize array data (HK1-5), a genomic DNA control (GDC), replicate reverse-transcription controls (RTC), and replicate positive PCR controls (PPC) was used. Custom RT^2^ Profiler PCR Arrays were performed on the StepOnePlus quantitative PCR cycler (Thermo Fisher Scientific, Waltham, MA, USA) using the RT^2^ SYBR Green qPCR Mastermix (Qiagen, Maryland, ME, USA), with a thermal cycling condition of 95 °C for 10 min followed by 40 cycles of 95 °C for 15 s and 60 °C for 1 min, followed by a melting curve acquisition step of 95 °C, 1 min; 65°C, 2 min (optics off); 65 °C to 95 °C at 2 °C/min. Briefly, cDNA synthesis reaction (20 µL per sample) was diluted in 91 µL of RNase-free water, and 102 μL of the aliquot was mixed with 1248 µL of RNase-free water and 1350 μL of 2 × RT^2^ SYBR green RT^2^ Mastermix. Then, 25 µL of the PCR components mix was added to each well of the RT^2^ Profiler PCR Array. Ct values of all samples were exported from the qPCR instrument and uploaded into GeneGlobe Data Analysis Center. Changes in the expression of gene of interest (GOI) normalized to that of the housekeeping gene (HKG) were analyzed using 2^−ΔΔC_T_^ method.

### 2.12. SYBR Green Real-Time Quantitative Polymerase Chain Reaction

Of the differentially expressed genes (DEGs) analyzed, the top 10 DEGs with the highest discrepancy levels from the controls were selected for further study. GAPDH was considered as a constitutive housekeeping gene. Specific primers and probes targeting these genes were used (Supplementary Table S2). SYBR green qPCR was performed on the StepOnePlus quantitative PCR cycler (Thermo Fisher Scientific, MA, USA) using the Thunderbird^TM^ SYBR qPCR Mix (Toyobo, Osaka, Japan), with a thermal cycling condition of 95 °C for 60 s followed by 40 cycles of 95 °C for 15 s and 60 °C for 60 s, followed by a melting curve acquisition step of 95 °C, 1 min; 65 °C, 2 min (optics off); 65 °C to 95 °C at 2 °C/min. Reaction mixture was setup as 10 µL thunderbird SYBR qPCR Mix, 6 *p*mol forward primer, 6 *p*mol reverse primer, 2 µL cDNA solution, 0.4 µL of 50X ROX reference dye, and 1.6 µL DDW. Fold changes were analyzed using 2^−ΔΔC_T_^ method. Briefly, (1) ΔC_T_ for on each array for each gene: ΔC_T_ = C_T_ (a target gene)—C_T_ (GAPDH), (2) average ΔC_T_ for each gene within a group, (3) ΔΔC_T_ for each gene between groups: ΔΔC_T_ = ΔC_T_ (a target sample)—ΔC_T_ (a reference sample), (4) fold change: 2^−ΔΔC_T_^ was determined.

### 2.13. Statistical Analysis

Statistical analysis was performed using GraphPad Prism, version 8.4.3 (GraphPad, San Diego, CA, USA), with a 5% level of significance and two-tailed p values. Values were presented as mean ± standard deviation (SD). Logarithmic transformation of the data was carried out to obtain an approximately normal distribution of the viral titers (PFU) and viral genome copy values, and data were tested for normal distribution using the Shapiro–Wilk test. Log_10_ transformed viral titers (PFU) and viral genome copy values were analyzed either in two-group or multiple-group comparisons. Two-group comparisons were analyzed using Student’s *t*-test. Multiple-group comparisons were analyzed by running both parametric (ANOVA) and non-parametric (Kruskal–Wallis) statistical tests with Dunn’s and Tukey’s post hoc tests. Differences in statistical significance were indicated with asterisks: single asterisk (*) indicates a p value of less than 0.05 (*p < 0.05); double asterisks (**) indicate a p value of less than 0.01 (** *p* < 0.01); triple asterisks (***) indicate a p value of less than 0.001 (*** *p* < 0.001); quadruple asterisks (****) indicate a p value of less than 0.0001 (**** *p* < 0.0001); and ns indicates a p value of over 0.05 (*p* > 0.05). Number of replicates per experiment is indicated in each figure legend.

## 3. Results

### 3.1. Optimizing Multiplicities of Infection (MOI) and Viral Supernatant Harvesting Time Points

To determine the optimal MOI capable of establishing persistent infecting and viral supernatant harvesting time point for viral progeny production, infection with three MOIs (0.1, 0.01 and 0.001) were performed in Vero 9013 cells. As shown in Supplementary Figure S1, for both JEV OH0566 and ZIKV PRVABC59, at the MOI of 0.1, 0.01, and 0.001, the virus infectivity reached its peak at around 48 h p.i., 72 h p.i., and 96 h p.i. respectively, and declined thereafter. However, the unbound virus was still detectable and produced a false-positive test result at an MOI of 0.1. For DENV-2 TL-30, all MOIs reached peak at around 96 h p.i. The viral titer peaks for the MOI of 0.1 was lowest. Infection with an MOI at 0.01 resulted in higher titers for all three virus strains. Accordingly, an MOI of 0.01 was used to infect confluent Vero 9013 cells for the following experiments: the endpoint was chosen at 72 h p.i. for ZIKV PRVABC59 and JEV OH0566 and 96 h p.i. for DENV-2 TL-30 respectively.

### 3.2. Antiviral Efficacy and Cytotoxicity in Vero 9013 Cells

**Figure 1:**
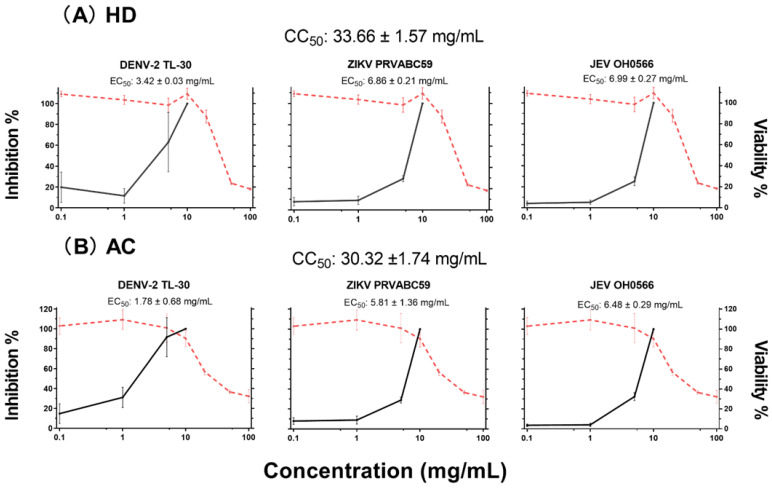
Herb extract cytotoxicity and dose–response relationships of antiviral efficacy. (1) Cytotoxicity (red lines): Vero 9013 cells were seeded at a density of 2 × 10^4^ cells/well and cultured for 24 h. Cells were overlaid with media containing various concentrations of each herb and cultured an additional 4 days. MTT cell proliferation and cytotoxicity assay was used to determine cell viability, which was normalized for CC_50_ determination. (2) Dose–response relationships of antiviral efficacy (black lines): Vero 9013 cells were incubated with each virus at MOI 0.01 for 1 h, and unbound virus was removed by washing with EMEM to synchronize the replication, then treated with each herb at a range of concentrations (10, 5, 1, and 0.1 mg/mL). Supernatants were collected to determine infectious viral titers at 72 h p.i. for JEV OH0566 and ZIKV PRVABC59 and 96 h p.i. for DENV-2 TL-30, respectively. Inhibition (%) = (viral titer (log_10_ PFU/mL) of treated culture supernatants/mean viral titer (log_10_ PFU/mL) of untreated viral-infected control culture supernatants) × 100%. The value of inhibition (%) was normalized for EC_50_ determination. The error bars represented the standard deviation of the mean of duodecuplicate (12 copies). (**A**) Cytotoxicity and viral titer analysis for which treatment with *Hedyotis diffusa* (HD). (**B**) Cytotoxicity and inhibition in *Artemisia capillaris*. (AC) treated samples.

Both HD and AC manifested low cytotoxicity to Vero 9013 with CC_50_ values of 33.66 ± 1.57 and 30.32 ± 1.74 mg/mL (mean ± SD). Both viabilities increased with decreasing concentration of herbs in a dose-dependent manner (Figure 1). Balancing between toxicity and viral replication inhibition, a range of concentrations less than or equal to 10 mg/mL of the herbs demonstrated low toxicity, with cells that were chosen for the subsequent antiviral experiments.

The antiviral activity of HD and AC was confirmed by measuring their treatment against viral infection at a range of non-cytotoxic concentrations (0.1 ~ 10 mg/mL) in Vero 9013 cells. After each virus adsorption at an MOI of 0.01, Vero 9013 cell monolayers were incubated in the absence or presence of increasing doses of HD or AC as indicated. Supernatants were collected to determine infectious viral titer via plaque assay at 72 h p.i. for JEV OH0566 and ZIKV PRVABC59 and 96 h p.i. for DENV-2 TL-30, respectively.

**Figure 2:**
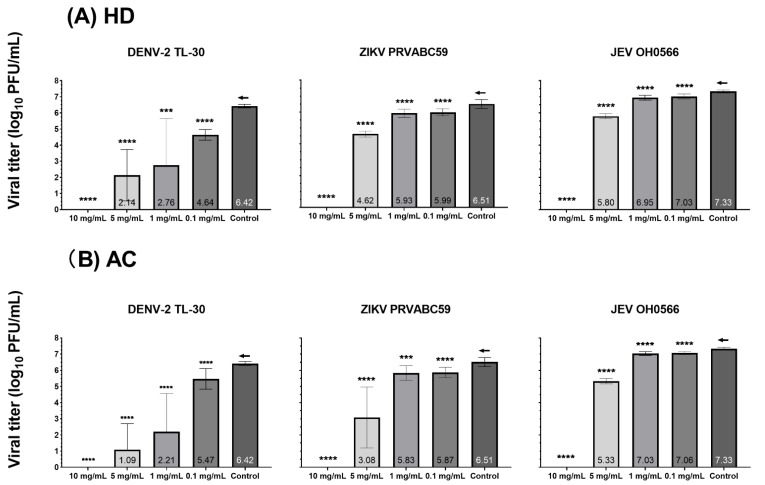
Inhibition effect of *Hedyotis diffusa* (HD) and *Artemisia capillaris* (AC) on DENV-2 TL-30, ZIKV PRVABC59, and JEV OH0566 viral replication via plaque reduction assay. Vero 9013 cells were incubated with each virus at MOI 0.01 for 1 h, and the unbound virus was removed by washing with EMEM to synchronize the replication, then treated with each herb at a range of concentrations (10, 5, 1, and 0.1 mg/mL). Supernatants were collected to determine infectious viral titers at 72 h p.i. for JEV OH0566 and ZIKV PRVABC59 and 96 h p.i. for DENV-2 TL-30, respectively. Viral titers are expressed as mean ± standard deviation log_10_ PFU/mL. (**A**) Viral titer analysis following HD treatment. (**B**) Inhibition in AC treated samples. Viral titers were titrated using Vero 9013 cells. All experiments were performed using duodecuplicate (12 copies). ←: control; *** *p* < 0.001; **** *p* < 0.0001; and ns indicates a *p* value of over 0.05 (*p* > 0.05).

In the presence of HD or AC at a concentration of 10 mg/mL, plaque formation was completely inhibited against all the tested virus strains (Figure 1, Figure 2 and Table S3.). At a concentration of 5 mg/mL, treatment with the herbs demonstrated a significant decrease in viral titer when compared to the untreated viral-infected control. Viral replication inhibition of HD treatment demonstrated lower viral titer number by 10-fold, with log10 PFU/mL differences of 1.5 ± 0.2 (*p* < 0.0001), 1.9 ± 0.2 (*p* < 0.0001), and 4.3 ± 1.6 (*p* < 0.0001) for JEV OH0566, ZIKV PRVABC59, and DENV-2 TL-30, respectively. Similarly, AC treatment inhibited viral replication by 10-fold, with a reduction of 2.0 ± 0.2 (*p* < 0.0001) for JEV OH0566, 3.4 ± 1.9 (*p* < 0.0001) for ZIKV PRVABC59, and 5.3 ± 1.6 (*p* < 0.0001) for DENV-2 TL-30, respectively.

At a concentration of 1 mg/mL, HD treatment significantly reduced the viral titer number by 10-fold compared those of untreated viral-infected control, with a log_10_ PFU/mL difference of 0.4 ± 0.2 (*p* < 0.0001) for JEV OH0566, 0.6 ± 0.3 (*p* < 0.0001) for ZIKV PRVABC59, and 3.7 ± 2.9 (*p* < 0.001) for DENV-2 TL-30, respectively. Similarly, AC treatment inhibited viral replication by 10-fold, with a log_10_ PFU/mL difference of 0.30 ± 0.2 (*p* < 0.0001) for JEV OH0566, 0.7 ± 0.5 (*p* < 0.001) for ZIKV PRVABC59, and 4.2 ± 2.4 (*p* < 0.0001) for DENV-2 TL-30, respectively.

At a concentration of 0.1 mg/mL, viral replication inhibition was also observed. As compared to the titers of untreated viral-infected control, HD treatment decreased viral titers by 10-fold, with a log_10_ PFU/mL difference of 0.3 ± 0.2 (*p* < 0.0001) for JEV OH0566, 0.5 ± 0.2 (*p* < 0.0001) for ZIKV PRVABC59, and 4.1 ± 2.4 (*p* < 0.0001) for DENV-2 TL-30, respectively. Similarly, AC treatment suppressed viral replication number by 10-fold, with a log_10_ PFU/mL difference of 0.3 ± 0.1 (*p* < 0.0001) for JEV OH0566, 0.6 ± 0.3 (*p* < 0.0001) for ZIKV PRVABC59, and 1.9 ± 2.2 (*p* < 0.01) for DENV-2 TL-30, respectively.

The antiviral potency and selectivity of HD and AC were evaluated by using plaque assays. The EC_50_, CC_50_, and selectivity index (SI) values of HD and AC are summarized in Table 1. Analysis of EC_50_ and SI values showed that HD and AC had high efficacy and safety margins against the three flavivirus strains. For HD treatment, the EC_50_ values of viral replication inhibition ranged from 3.4 ± 0.03 mg/mL for DENV to 7.0 ± 0.3 mg/mL for JEV OH0566. For AC treatment, they ranged from 1.78 ± 0.68 mg/mL for DENV-2 TL-30 to 6.5 ± 0.3 mg/mL for JEV OH0566, and were dependent on the species of flavivirus tested. The results revealed that HD and AC had the highest SI against DENV-2 TL-30, with values of 9.8 and 18.9, respectively.

These results suggested that HD and AC inhibited JEV OH0566, ZIKV PRVABC59, and DENV-2 TL-30 plaque formation at concentrations with limited cytotoxicity. As HD and AC inhibited plaque formation, the results suggest that the herbs could inhibit virus propagation.

### 3.3. Quantification of Viral RNA Genome Copy by RT-qPCR

To determine the antiviral activity of HD and AC viral genomic RNA in the culture supernatant of herb-treated and untreated cells, infected Vero cells were measured by RT-qPCR. HD and AC significantly inhibited all the tested virus strains with lower viral RNA genome copies by ten-fold in the supernatants at concentrations of 5 and 10 mg/mL (Table 2.), as compared to the untreated viral-infected control.

**Figure 3:**
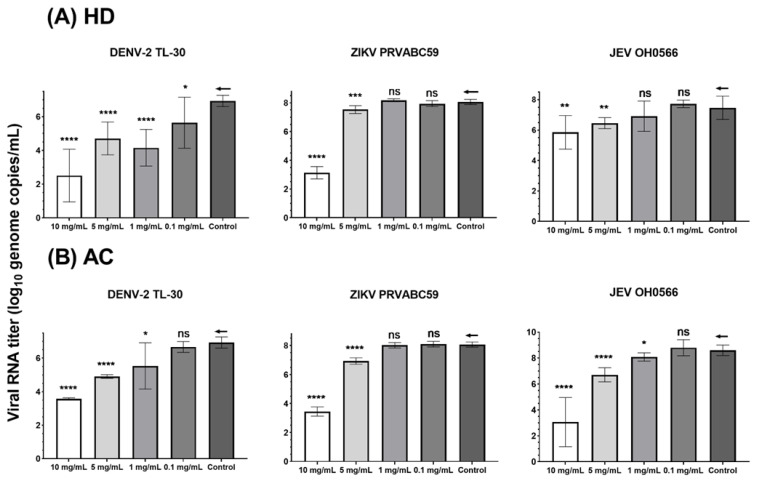
Viral genomic RNA levels of DENV-2 TL-30, ZIKV PRVABC59, and JEV OH0566 treated with *Hedyotis diffusa* (HD) and *Artemisia capillaris* (AC) in Vero 9013 cells as determined by RT-qPCR. Supernatants were collected to determine extracellular viral load at 72 h p.i. for JEV OH0566 and ZIKV PRVABC59 and 96 h p.i. for DENV-2 TL-30, respectively. The viral RNA levels were quantified by RT-qPCR as log_10_ viral genome copies/mL. (**A**) viral RNA level analysis following HD treatment. (**B**) Inhibition in AC treated samples. The error bars represented the standard deviation of the mean of octuplicate (8 copies); ←: control; * *p* < 0.05; ** *p* < 0.01; *** *p* < 0.001; **** *p* < 0.0001; and ns indicates a *p* value of over 0.05 (*p* > 0.05).

Significant (1.3 ± 1.5 (*p* < 0.05) and 2.8 ± 1.1 (*p* < 0.0001) log_10_ viral RNA copies/mL (mean ± SD)) reductions in viral RNA titer were observed in HD-treated DENV-2 TL-30 infected cells at concentrations of 1 and 0.1 mg/mL. However, HD did not significantly suppress the viral RNA titer of JEV OH0566 and ZIKV PRVABC59 at concentrations of 1 and 0.1 mg/mL (Figure 3.).

AC treatment (1 mg/mL) inhibited growth of DENV-2 TL-30 and JEV OH0566, with a log_10_ viral RNA copies/mL difference of 1.4 ± 1.4 (*p* < 0.05) and 0.9 ± 0.5 (*p* < 0.05), respectively. However, AC (1 mg/mL) did not reduce the ZIKV PRVABC59 viral RNA level. At a concentration of 0.1 mg/mL, AC treatment did not induce a significant reduction in viral RNA level against all the tested virus strains. While there was no statistical significance, viral RNA was reduced by 10-fold genomic copies. As compared to the untreated viral-infected control, viral RNA titers had an inverse association with HD and AC concentrations in a dose-dependent manner. This finding indicates that HD and AC effectively inhibit JEV OH0566, ZIKV PRVABC59, and DENV-2 TL-30 replication in vitro.

### 3.4. Time of Drug Addition

**Figure 4:**
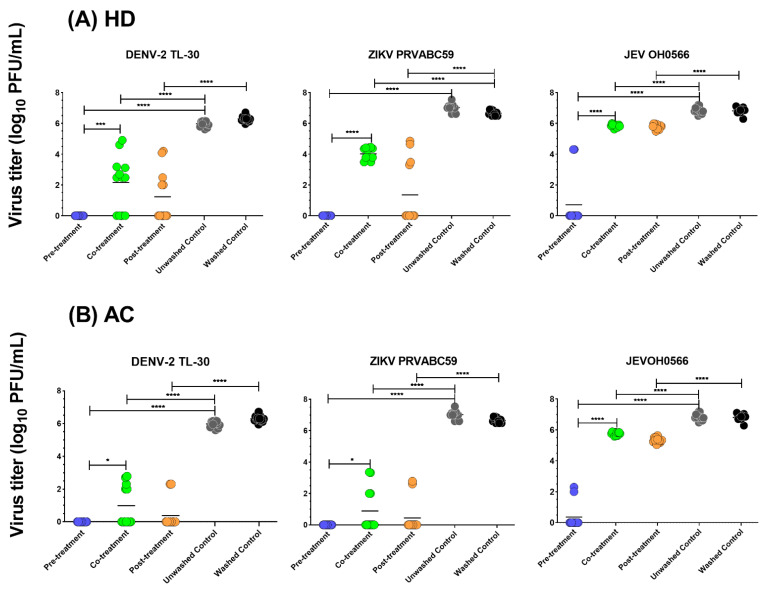
Efficacy of *Hedyotis diffusa* (HD) and *Artemisia capillaris* (AC) at 5 mg/mL concentration against JEV, ZIKV, and DENV-2 at an MOI of 0.01 in Vero cells, as quantified by plaque assay in pre-, co-, and post-treatment studies. Log_10_ transformed viral titer values of herb-treated viral-infected cell culture supernatants were compared with that of the untreated viral-infected control. (1) Pre-treatment process (−1 h p.i.): Vero cells were treated with herbs at a concentration of 5 mg/mL at 1 h prior to viral infection; (2) co-treatment process (0 h p.i.): Vero were treated with herbs at 5 mg/mL during virus infection; (3) post-treatment process (1 h p.i.): Vero 9013 cells were infected with virus for 1 h, viral inoculum was aspirated, cells were washed with EMEM twice and replaced with EMEM with 10% FBS. (**A**) viral titer analysis following HD treatment. (**B**) Inhibition in AC treated samples. The error bars represent the standard deviation of the mean of 12 replicates; * *p* < 0.05; *** *p* < 0.001; **** *p* < 0.0001.

To further evaluate the time point of infection by which the herbs exhibited antiviral activity against flavivirus infection, the time of drug addition study was conducted. In the pre-treatment study, at a non-cytotoxic concentration (5 mg/mL) of HD and AC, no infectious virus particles in the cell culture supernatant were detected for ZIKV and DENV-2 strains. In comparison with the untreated viral-infected control, pre-treatment of HD inhibited JE viral growth (89.5% log reduction value (LRV) (6.1 ± 1.7 log_10_ PFU/mL, *p* < 0.001)). Similarly, pre-treatment of AC also demonstrated an inhibitory activity of 94.8% LRV (6.5 ± 0.8 log_10_ PFU/mL, *p* < 0.001) on JEV (Figure 4). A similar significant reduction pattern of infection for HD and AC against all the three virus strains was observed in co- and post-treatment processes (*p* < 0.0001). Post-treatment of the herbs also significantly suppressed all the viral replication (*p* < 0.0001). Compared with viral plaque in the co-treatment group, HD pre-treatment significantly reduced viral titers, with a log_10_ PFU/mL difference of 5.1 ± 0.2 (*p* < 0.001) for JEV. Similarly, the viral titers of AC pre-treatment were significantly lower that of co-treatment, with a log_10_ PFU/mL difference of 5.4 ± 0.9 (*p* < 0.001) for JEV. Co-treatment of HD or AC exhibited anti-ZIKV and DENV-2 activities; by comparison, pre-treatment showed significantly higher (*p* < 0.001) and absolute inhibition of viral plaque formation. Although co- and post-treatment of the herbs showed strong inhibition of the tested viral replication, pre-treatment demonstrated comparatively higher antiviral efficacy (Table 3).

Inhibition of HD and AC at 5 mg/mL concentration against JEV OH0566, ZIKV PRVABC59, and DENV-2 TL-30 at an MOI of 0.01 in Vero cells was quantified by plaque assay in pre- co-, and post-treatment processes. Log_10_ transformed viral titer values of herb-treated viral-infected cell culture supernatants were compared with that of the untreated viral-infected control. The inhibition was calculated as follows: inhibition (%) = (viral titer (log_10_ PFU/mL) of treated viral-infected cell culture supernatants/mean viral titer (log_10_ PFU/mL) of untreated viral-infected cell culture supernatants) ×100%.

In this study, pre-treatment of the herbs significantly reduced viral titers in the cell culture supernatant among all the methods tested. Additionally, both herbs strongly inhibited infectious progeny replication in all the tested virus strains, regardless of the time of drug addition.

### 3.5. Antiviral Activity of HD and AC in Human-derived Cell Lines

**Figure 5:**
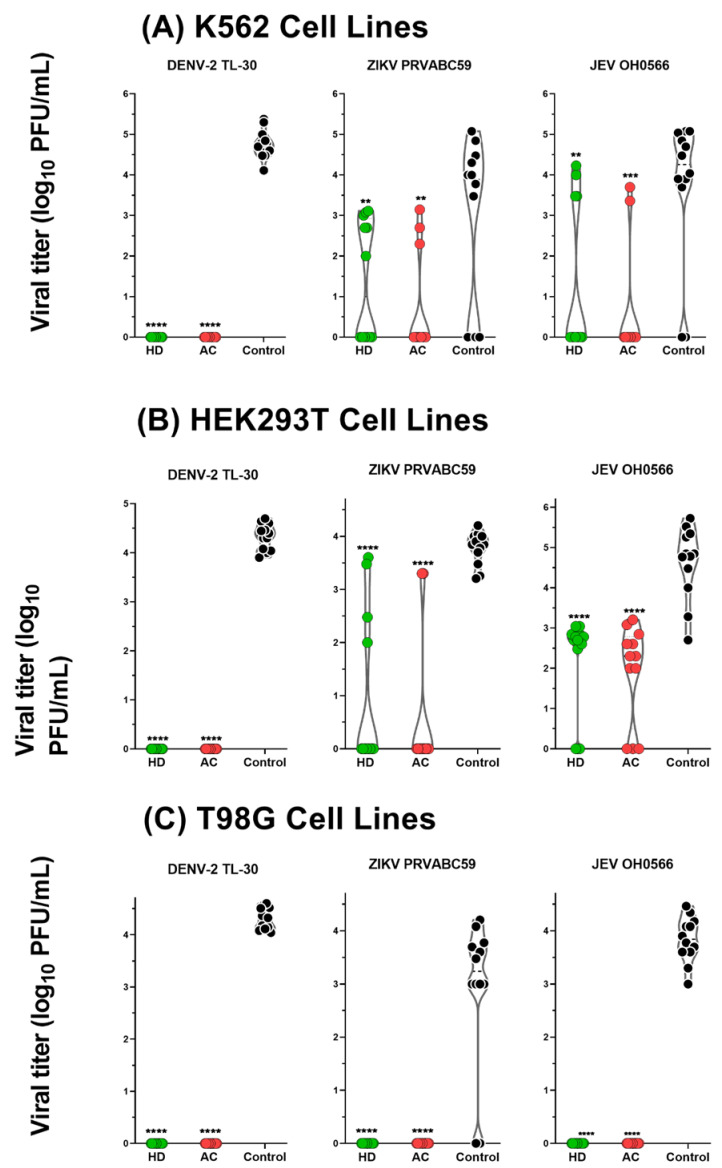
Antiviral activity in human cells. Cells were treated with HD or AC at a concentration of 5 mg/mL. (**A**) K562 cells were infected with JEV OH0566 at MOI 0.01 and ZIKV PRVABC59 and DENV-2 TL-30 at MOI 0.5; (**B**) T98G cells were infected with each virus at MOI 0.5; (**C**) HEK293T cells were infected with JEV OH0566 at MOI of 0.5 and ZIKV PRVABC59 and DENV-2 TL-30 at MOI of 1. Supernatants were collected at 72 h p.i. for JEV OH0566 and ZIKV PRVABC59 and 96 h p.i. for DENV-2 TL-30. Viral titers were determined by plaque assay. The error bars represented the standard deviation of the mean of duodecuplicate (12 copies); ** *p* < 0.01; *** *p* < 0.001; **** *p* < 0.0001. HD: *Hedyotis diffusa*; AC: *Artemisia capillaris*.

To determine the antiviral activity in human cells, K562, T98G, and HEK293T cell lines were used in this experiment. In the K562 cells culture supernatant, HD and AC treatment significantly reduced JEV OH0566 replication at a log reduction value of 65.9% (2.5 ± 1.88, *p* < 0.01) and 84.2% (3.1 ± 1.4, *p* < 0.001) when compared to the untreated viral-infected control: 51.2% (1.4 ± 1.5, *p* < 0.01) and 76.0% (2.2 ± 1.2, *p* < 0.01) LRV reduction against ZIKV PRVABC59 (Figure 5).

Of note, in the T98G cell culture supernatant, HD and AC treatment completely (100%, *p* < 0.0001) inhibited the infectious progeny production against all the tested virus strains. In the HEK293T cells culture supernatant, HD and AC treatment also significantly reduced JEV OH0566 replication at a log_10_ reduction value of 49.9% (2.3 ± 1.1, *p* < 0.0001) and 58.7% (2.7 ± 1.2, *p* < 0.0001) when compared to the untreated viral-infected control: 74.5% (2.81 ± 1.48, *p* < 0.0001) and 85.4% (3.2 ± 1.3, *p* < 0.0001) reduction against ZIKV PRVABC59. Notably, the herbs exhibited 100% (*p* < 0.0001) inhibition against DENV-2 TL-30 infection in all the used human cells.

In the K562, T98G, and HEK293T cell lines, significant reduction in viral titers following the herb treatments were demonstrated; however, the inhibition level of viral replication was higher in T98G cell lines in comparison to the other two human cells. Therefore, this experiment confirmed the antiviral potency of HD and AC in human cells against JEV, ZIKV, and DENV-2 infection.

### 3.6. Screening of Differentially Expressed Genes (DEGs)

Total RNA isolated from T98G cells at 6 h post treatment of the herbs against DENV-2 TL-30 strain (MOI = 1) infection were analyzed by using RT^2^ Profiler PCR array to evaluate the expression of 84 genes associated with human inflammatory cytokines and receptors (Supplementary Table S4). Comparative analysis of the gene expression profile demonstrated the broadest dysregulation of genes in the number of differentially expressed genes (DEGs), as shown in Supplementary Figure S2. The top ten DEGs were then selected for further studies.

### 3.7. Herbs Altered Gene Expression Profile Response to Viral Infection

In the next series of experiments, the selected DEGs were validated by SYBR Green real-time PCR (Table 4). Viral-infected or uninfected T98G cells were treated or untreated with herbs, and their proliferation potentials were analyzed at 6 min (m p.t.) and 6 h (h p.t.) post-treatment. The experimental data were gathered from the following five pairwise comparisons: (1) viral-infected cells vs. cells, (2) herb-treated viral-infected cell vs. cells, (3) herb-treated cells vs. cells, (4) herb-treated viral-infected cells vs. herb-treated cells, and (5) herb-treated viral-infected cells vs. viral-infected cells.

The changes induced by DENV-2 TL-30 infection were investigated by the pairwise comparison analysis between infected cells and uninfected cells. A total of five cytokines/chemokines were elevated (range: 1.4- to 7.8-fold) and two were repressed (range: 0.6- to 3.1-fold) at 6 m p.t., and one was elevated (1.7-fold) and two repressed (1.3- and 1.9-fold, respectively) (Supplementary Figure S3.) at 6 h p.t. As compared to the profile displayed by the uninfected cells, a few genes were reduced or enhanced the alteration by viral infection following herb treatment upon HD treatment: CCL5 (up 2.5-fold, *p* = 0.0004, 6 m p.t.; down 1630.1-fold, *p* = 0.0014, 6 h p.t.), BMP2 (up 1.9-fold, *p* = 0.03, 6 m p.t.), CCL26 (down 1.3-fold, *p* = 0.013, 6 h p.t.), and CCL15 (up 1.5-fold, *p* = 0.04, 6 m p.t.; 1.3-fold, *p* = 0.04, 6 h p.t.). Regarding exposure to AC, the results are the following: IL21 (up 1.4-fold, *p* = 0.009, 6 m p.t.), IL27 (up 1.9-fold, *p* = 0.005, 6 m p.t.), CCR1 (up 5.3-fold, *p* = 0.04, 6 m p.t.), and CCL15 (up 2.1-fold, *p* < 0.0001, 6 h p.t.) (Figure S4.). However, the majority of the tested genes reversed the expression profiles displayed by uninfected cells by a statistically significant degree following herb treatment in the viral-infected cells. Uninfected cells were exposed to herbs and analyzed in parallel to exclude gene expression change induced by the direct effect of herb treatment. As compared to untreated cells, HD treatment repressed CCL26 (3.7-fold, *p* = 0.0006) and CCL5 (1.3-fold, *p* = 0.015) at 6 m p.t and 6 h p.t., respectively (Figure S5). The expression of major genes tested were elevated (range 15.6–68.2-fold) upon AC treatment at 6 m p.t., except CCL5 and CXCL13; however, uninfected cells showed no significant effect of herbs on the gene expression at 6 h p.t.

To control for non-virus-specific immunomodulatory effects of herbs, the changes in gene expression were analyzed between herb-treated viral-infected and treated uninfected cells. Compared to the herb response in uninfected cells, several genes following HD treatment showed small changes at 6 m p.t.: IL17C (down 2.2-fold, *p* = 0.04), CCL5 (up 6.1-fold, *p* = 0.03), CCL15 (up 2.1-fold, *p* = 0.0008), BMP2 (up 2.1-fold, *p* = 0.03), IL27 (up 2.0-fold, *p* = 0.004). In addition, IL21 was down 1.4-fold (*p* = 0.005) at 6 h p.t. (Figure S6). Upon AC treatment, the majority of gene expressions were largely decreased (range: 16.4- to 86.6-fold) at 6 m p.t., and increased little (IL21: 1.5-fold, *p* = 0.002; CCL15: 1.7-fold, *p* = 0.02) at 6 h p.t. Relatively few gene expressions were similar in viral-infected and uninfected cells following herb treatment. Therefore, the gene expression differences were detected after herb treatment in viral-infected cells, and their uninfected counterparts were associated with the human response to viral infection.

**Figure 6:**
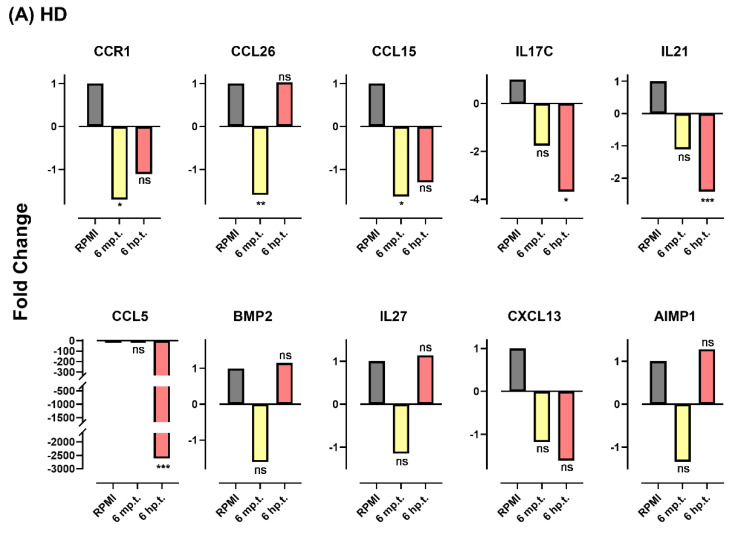

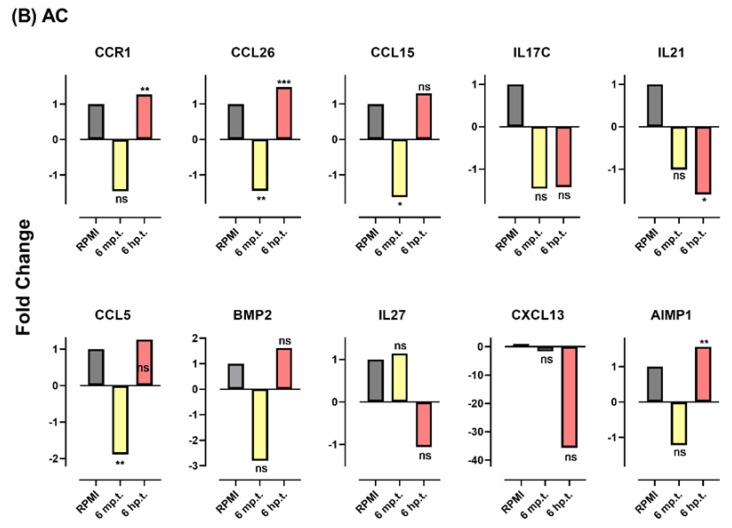
The fold changes of the selected inflammatory genes in herb-treated DENV-2 TL-30 infected T98G cells relative to untreated viral-infected cells. T98G cells were incubated with DENV-2 TL-30 at MOI = 1 for 1 h, and the unbound virus was removed by washing with RMPI to synchronize the replication, then treated with each herb at a concentration of 5 mg/mL or RPMI supplemented with 15% FCS. Cells were collected to determine gene expression level at 6 min and 6 h post-treatment. (**A**) Selected genes for which treatment with *Hedyotis diffusa* (HD); (**B**) gene expression in *Artemisia capillaris* (AC) treated samples; * *p* < 0.05; ** *p* < 0.01; *** *p* < 0.001; and ns indicates a *p* value of over 0.05 (*p* > 0.05).

To assess the effect of antiviral treatment, pairwise comparison analyses of herb-treated and untreated viral-infected cells were undertaken. Compared to untreated viral-infected cells, during the early stages of HD treatment (6 m p.t) in viral-infected cells, CCR1 (1.7-fold, *p* = 0.01), CCL26 (1.6-fold, *p* = 0.004), and CCL15 (1.6-fold, *p* = 0.02) were repressed (Figure 6, Table 4); CCL5 (2615.1-fold, *p* = 0.0006), IL21 (2.4-fold, *p* = 0.0002), and IL17C (3.7-fold, *p* = 0.03) were downregulated at 6 h p.t. CCL5 displayed the greatest repressed expression levels. Upon AC treatment, compared to untreated samples, CCL5 (1.9-fold, *p* = 0.002), CCl26 (1.45-fold, *p* = 0.004), and CCL15 (1.6-fold, *p* = 0.02) were repressed at 6 m p.t.; IL21 (1.6-fold, *p* = 0.02) was also repressed at 6 h p.t. Conversely, CCR1 (1.3-fold, *p* = 0.008), CCL26 (1.5-fold, *p* = 0.0003), and AIMP1(1.6-fold, *p* = 0.002) demonstrated elevated expression levels following AC treatment in viral-infected cells.

Multiple comparison analysis indicated that up/downregulated genes by viruses in infected cells relative to their uninfected counterparts demonstrated statistically significant reversal in viral-infected cells following herb treatment (Figure 6, and Figure S3). Elevated cytokines and receptors including CCL26, CCL15, and CCR1 were responsive to statistically significant reversal following exposure of infected cells to HD for 6 min. CCL15 was also significantly reversed upon AC treatment for 6 min.

## 4. Discussion

Medicinal plants have historically been valuable sources of potential therapeutic products and continue to be an attractive source of discovery of novel antiviral compounds [51]. While *Hedyotis diffusa* (HD) and *Artemisia capillaris* (AC) have been identified in TCM to possess “heat-clearing” and detoxifying effects, there is limited scientific evidence of the antiviral potential of HD and AC against *Flaviviridae*. Since Vero cells are highly susceptible to a variety of viruses and are incapable of producing any type I interferons, they have been proven to be the first-choice cell model for various types of life-threating emerging viral pathogens [52,53]. In this study, HD and AC were used to determine the utility in viral replication inhibition by using an in vitro model of infection.

A selectivity index of at least four has been recommended as an index with good antiviral selectivity [54,55]. In this context, HD and AC demonstrated SI values of >4, indicating that the herbs are potentially useful antivirals for JEV, DENV, and ZIKV. Time of drug addition assays with the herbs against various virus strains were performed to maximize the possibility of antiviral efficacy. Compared with the co-treatment (0 h p.i.), the addition of the herbs markedly inhibited infectious progeny production following pre-treatment (−1 h p.i.) against all the tested virus strains. The greatest inhibition of virus attachment was inhibited with the addition of the herbs at the 1 h earlier time points (−1 h p.i.) than when they were added at 0 h p.i. (Figure 4). The finding indicated that the herbs may suppress the virus uptake and inhibit the viral attachment to cells in the initial step in the infection process. HD and AC inhibition of viruses by the co-treatment process indicated the virucidal potential. On the other hand, HD and AC significantly reduced the viral titers by post-treatment process, indicating that both herbs may suppress the infectious progeny replication. This data suggests that the antiviral efficacy of HD and AC by acts by inhibiting viral attachment, entry, and replication, with the potential of involvement of an array of immunological cascades in each phase of the viral infection cycle.

Apart from Vero cells, which is a cell line that is commonly used in flavivirus propagation assays, various human cell lines (K562, HEK293T, and T98G) were used to investigate the antiviral activity of HD and AC. Viral growth inhibition was observed in the three cell lines infected with all the tested virus strains in the presence of herb treatment. Notably, the addition of HD and AC herbs resulted in 100% inhibition of viral plaque formation against all the tested virus strains in T98G cells (Figure 5). After entering the CNS, neurotropic flaviviruses can infect neurovascular unit cells such as astrocytes, and lead to general neuroinflammation and blood–brain barrier (BBB) impairment [56,57]. Astrocytes are mediators of neuroinflammation, and these infections may further disrupt neuronal activity [58,59]. Numerous neurotropic flaviviruses reportedly target astrocytes, such as DENV [60], ZIKV [61,62], and JEV [63]. T98G cell lines have been proven useful as a human astrocyte model, which have similar morphological and functional properties in comparison to other human astrocytes [64]. In this context, T98G was chosen as a model cell line for DENV-2 infection. These results suggest that HD and AC may represent a novel therapeutic agent to mitigate CNS manifestations of viral infection.

Acute flavivirus infections are characterized by extensive inflammation and chemokine expression, which is associated with outcomes including enhanced viral dissemination, tissue damage, and viral burden [65,66]. Downregulated expression during HD treatment in viral-infected cells as compared to their untreated viral-infected counterparts includes CCR1, CCL26, and CCL15 at 6 m p.t, and CCL5, IL21, and IL17C at 6 h p.t. Upon AC treatment, compared to untreated viral-infected samples, CCL5, CCl26, IL21, and CCL15 levels were decreased at 6 m p.t. In contrast, CCR1, CCL26, and AIMP1 levels were elevated following AC treatment in viral-infected cells. Of the genes demonstrating downregulated expression level in HD-treated viral-infected cells, chemokine CCL5 (Figure 6) expression was significantly lower. C-C motif chemokine ligand 5 (CCL5) has been identified as a chemokine that is localized in white matter tracts undergoing demyelination following viral infection. The chemokine has been hypothesized to participate in viral pathogenesis by attracting activated leukocytes and activated macrophages into the CNS, and in turn, leading to neurological impairment. Selective neutralization of CCL5 resulted in diminished leukocyte infiltration into the CNS and reduced neurological disability in a viral model of multiple sclerosis [67]. The cytokine CCR1 had a role in the development of detrimental pulmonary responses during respiratory syncytial virus (RSV), and the absence of CCR1 may lead to lead to cellular damage and/or altered immune activation [68]. In contrast, CCL15 was significantly associated with the expression of Hepatitis B virus X protein and negatively correlated with clinical outcome for HBV-positive hepatocellular carcinoma (HCC) patients [69,70]. Overall, together with inhibiting virus propagation, the cytokine signature suggests that the HD and AC could potentially alter the expression of proinflammatory cytokines during viral infection, which in turn results in alleviating pathogenicity in the target tissue.

## 5. Conclusions

*Hedyotis diffusa* (HD) and *Artemisia capillaris* (AC) inhibited flaviviral replication and potentially limit the inflammatory response generated by DENV infection. The results suggest that HD and AC should be viable candidates for further in vivo study to demonstrate their efficacy against viral infection and pathogenesis. Our results indicate that the herbs have efficacies as potential agents for prophylaxis or treatment of flavivirus infection

## Figures and Tables

**Table 1:** Representation of the antiviral activities of HD and AC by SI *.

Virus Strain	HD				CA		
	Vero9013 Cells	CC_50_ (mg/mL)	SI		Vero9013 Cells	CC_50_ (mg/mL)	SI
	EC_50_ (mg/mL)				EC_50_ (mg/mL)		
JEV OH0566	7.0 ± 0.3	33.7 ± 1.6	4.8		6.5 ± 0.3	30.3 ±1.7	4.7
ZIKV PRVABC59	6.9 ± 0.2		4.9		5.8 ± 1.4		5.2
DENV-2 TL-30	3.4 ± 0.03		9.8		1.8 ± 0.7		18.9

* Results are shown as mean ± SD. EC_50_: concentration required to inhibit 50% of virus replication; CC_50_: herb concentration that reduces the cell growth by 50% (cellular toxicity); selectivity index (SI) = CC_50_/EC_50_. HD: *Hedyotis diffusa*; AC: *Artemisia capillaris*.

**Table 2:** Reduction in viral genomic RNA level upon herb treatment at concentrations of 10 and 5 mg/mL.

Concentration	HD									AC							
	JEV OH0566			ZIKV PRVABC59			DENV-2 TL-30			JEV OH0566			ZIKV PRVABC59			DENV-2 TL-30	
	RNA Titer	*p* Value		RNA Titer	*p* Value		RNA Titer	*p* Value		RNA Titer	*p* Value		RNA Titer	*p* Value		RNA Titer	*p* Value
10 mg/mL	1.6 ± 1.1	0.004		4.9 ± 0.4	<0.0001		4.4 ± 1.6	<0.0001		5.5 ± 1.9	<0.0001		4.6 ± 0.31	<0.0001		3.3 ± 0.06	<0.0001
5 mg/mL	1.0 ± 0.3	0.005		0.5 ± 0.3	0.0004		2.2 ± 1.0	<0.0001		1.9 ± 0.5	<0.0001		1.1 ± 0.22	<0.0001		2.0 ± 0.1	<0.0001
1 mg/mL	NS **			NS			2.8 ± 1.1	<0.0001		0.9 ± 0.5	0.01		NS			1.4 ± 1.4	0.02
0.1 mg/mL	NS			NS			1.3 ± 1.5	0.0337		NS			NS			NS	

** NS: no significant differences. HD: *Hedyotis diffusa*; AC: *Artemisia capillaris*.

**Table 3:** Inhibition of HD and AC on viral replication in pre-, co-, and post-treatment processes.

	HD				AC		
	Pre-Treatment	Co-Treatment	Post-Treatment		Pre-Treatment	Co-Treatment	Post-Treatment
DENV-2 TL-30	100.0 ± 0.0	63.8 ± 29.7	80.4 ± 26.4		100.0 ± 0.0	83.6 ± 20.6	93.9 ± 14.2
ZIKV PRVABC59	100.0 ± 0.0	42.8 ± 5.6	79.6 ± 30.8		100.0 ± 0.0	87.3 ± 19.6	93.3 ± 15.8
JEV OH0566	89.5 ± 24.5	14.5 ± 1.5	14.8 ± 2.4		94.8 ± 12.3	15.5 ± 1.5	21.7 ± 2.4

**Table 4:** Determination of the selected inflammatory genes confirmed the up/downregulated expression in herb-treated DENV-2 TL-30 infected T98G cells compared to untreated viral-infected cells.

Symbol	Accession Number	Entrez Gene Name	Treatment Time										
			6 m p.t.			6 h p.t.			6 m p.t.			6 h p.t.	
			Fold Change	*p* Value		Fold Change	*p* Value		Fold Change	*p* Value		Fold Change	*p* Value
BMP2	NM_001200	Bone morphogenetic protein 2	NC *			NC			NC			NC	
IL17C	NM_013278	Interleukin 17C	NC			−3.7	0.03		NC			NC	
IL27	NM_145659	Interleukin 27	NC			NC			NC			NC	
IL21	NM_021803	Interleukin 21	NC			−2.4	0.0002		NC			−1.59	0.01
CCL5	NM_002985	C-C motif chemokine ligand 5	NC			−2615.2	0.0006		−1.89	0.002		NC	
CCR1	NM_001295	C-C motif chemokine receptor 1	−1.7	0.015		NC			NC			+1.27	0.008
CXCL13	NM_006419	C-X-C motif chemokine ligand 13	NC			NC			NC			NC	
AIMP1	NM_004757	Aminoacyl tRNA synthetase complex interacting multifunctional protein 1	NC			NC			NC			+1.6	0.001
CCL26	NM_006072	C-C motif chemokine ligand 26	−1.59	0.004		NC			−1.45	0.004		+1.5	0.0003
CCL15	NM_032964	C-C motif chemokine ligand 15	−1.63	0.02		NC			−1.63	0.02		NC	

* NC, no significant change; 6 m p.t.: 6 min post-treatment; 6 h p.t.: 6 h post-treatment. The fold changes in herb-treated DENV-2 TL-30 infected cells relative to untreated viral-infected cells are indicated: + upregulated; −downregulated. HD: *Hedyotis diffusa*; AC: *Artemisia capillaris*.

## Data Availability

The dataset is available upon reasonable request to the corresponding author.

## Supplementary Materials

The following supporting information can be downloaded at: https://www.mdpi.com/article/10.3390/plants11192589/s1, Table S1: Oligonucleotide primers and fluorogenic probes used in the JEV, ZIKV, and DENV-2 real-time RT-qPCR assays, Table S2: Oligonucleotide primers of the selected inflammatory genes used in SYBR green qPCR, Table S3: Antiviral efficacy of herbs in Vero 9013, Figure S1: Time-dependent viral titer of Vero 9013 cells inoculated with JEV, ZIKV, and DENV-2 at different virus concentrations, Figure S2: Relative expression comparison of 84 genes involved in human inflammatory cytokines and receptors, Figure S3: The fold changes of the selected inflammatory genes in DENV-2 TL-30 infected T98G cells relative to T98G cells, Figure S4: The fold changes of the selected inflammatory genes in herb-treated DENV-2 TL-30 infected T98G cells relative to T98G cells, Figure S5: The fold changes of the selected inflammatory genes in herb-treated T98G cells relative to T98G cells, Figure S6: The fold changes of the selected inflammatory genes in herb-treated DENV-2 TL-30 infected T98G cells relative to herb-treated uninfected cells.

## Abbreviations

AC	*Artemisia capillaris*
AIMP1	Aminoacyl tRNA synthetase complex interacting multifunctional protein 1
BMP2	Bone morphogenetic protein 2
CC50	Half maximal cytotoxic concentration
CCL15	C-C motif chemokine ligand 15
CCL26	C-C motif chemokine ligand 26
CCL5	C-C motif chemokine ligand 5
CCMG	Concentrated Chinese Medicine Granules
CCR1	C-C motif chemokine receptor 1
CNS	Central Nervous System
CXCL13	C-X-C motif chemokine ligand 13
DEGs	Differentially expressed gene
DENV	Dengue virus
DMEM	Dulbecco’s Modified Eagle Medium
EC50	Half maximal effective concentration
EMEM	Eagle’s minimum essential medium
FCS	Fetal calf serum
GDC	Genomic DNA control
h p.i.	Hour (s) post-infection
h p.t.	Hour (s) post-treatment
HD	*Hedyotis Diffusa*
HEK-293T cell lines	Human embryonic kidney cell lines
HKG	Housekeeping gene
IL17C	Interleukin 17C
IL21	Interleukin 21
IL27	Interleukin 27
JEV	Japanese encephalitis virus
K562 cell lines	Human chronic myeloid leukemia cell lines
LRV	Log reduction value
m p.t.	Minute (s) post the treatment
MOI	Multiplicities of infection
OD	Optical density
PFU	Plaque-forming units
PPC	Positive PCR control
RPMI	Roswell Park Memorial Institute
RT-qPCR	Quantitative real-time polymerase chain reaction
SI	Selectivity index
SYBR green qPCR	SYBR green real-time quantitative polymerase chain reaction
T98G cell lines	Human glioblastoma cell lines
TCM	Traditional Chinese medicine
ZIKV	Zika virus
