# Supplementary material for: Novel Antiviral Efficacy of Hedyotis diffusa and Artemisia capillaris Extracts against Dengue Virus, Japanese Encephalitis Virus, and Zika Virus Infection and Immunoregulatory Cytokine Signatures
Source: Plants (Basel). 2022 Sep 30;11(19):2589. doi: 10.3390/plants11192589 (PMC9571899; doi:10.3390/plants11192589)
Supplement: Supplementary file 1 [file plants-11-02589-s001.zip › plants-1863711-supplementary.pdf]

Table S1. Oligonucleotide primers and fluorogenic probes used in the JEV, ZIKV, and DENV-2 real-time RT-qPCR assays.

Virus	Primer and probes	Nucleotide sequence (5′ à 3′)	Target gene
	Forward Primer	CTGGAYTGTGARCCAAGGA	
JEV	Reverse Primer	GAHCCCACGGTCATGA	E
	Probe	FAM-ACTRAACACTGAAGCGT-MGB	
	Forward Primer	CCGCTGCCCAACACAAG	
	Reverse Primer	CCACTAACGTTCTTTTGCAGACAT	
ZIKV	Probe	FAM-AGCCTACCTTGACAAGCAGTCAGACACTCAA-BHQ	E
	Forward Primer	ACACCACAGAGTTCCATCACAGA	
DENV-2	Reverse Primer	CATCTCATTGAAGTCNAGGCC	E
	Probe	FAM- CGATGGARTGCTCTC-BHQ	

Table S2. Oligonucleotide primers of the selected inflammatory genes used in SYBR green qPCR

Gene	Accession Number	Primer	Nucleotide sequence (5′ à 3′)
AIMP1	NM_004757	Forward Primer	GCAGGCAACTTTGAGGGAAGAG
		Reverse Primer	TTAGCGTGCAGTGGAGTACCAG
BMP2	NM_001200	Forward Primer	TGTATCGCAGGCACTCGGGTCA
		Reverse Primer	CCACTCGTTTCTGGTAGTTCTTC
CCL15	NM_032964	Forward Primer	TGATGTCAAAGCTTCCACTGGAAA
		Reverse Primer	GAGTGAACACGGGATGCTTTGTG
CCL26	NM_006072	Forward Primer	GGGAGTGACATATCCAAGACCTG
		Reverse Primer	CAGACTTTCTTGCCTCTTTTGGTA
CCL5	NM_002985	Forward Primer	CCTGCTGCTTTGCCTACATTGC
		Reverse Primer	ACACACTTGGCGGTTCTTTCGG
CCR1	NM_001295	Forward Primer	CAACTCCGTGCCAGAAGGTGAA
		Reverse Primer	GTTCAGGAGGTAGATGCTGGTC
CXCL13	NM_006419	Forward Primer	TATCCCTAGACGCTTCATTGATCG
		Reverse Primer	CCATTCAGCTTGAGGGTCCACA
IL17C	NM_013278	Forward Primer	GCCCTCAGCTACGACCCAGTG
		Reverse Primer	AGCTTCTGTGGATAGCGGTCCT
IL21	NM_021803	Forward Primer	CCAAGGTCAAGATCGCCACATG
		Reverse Primer	TGGAGCTGGCAGAAATTCAGGG
IL27	NM_145659	Forward Primer	AGACGGCAGGCGACCTTG
		Reverse Primer	GCGAGATGCAGGCTGACTG
GAPDH	NM_002046	Forward Primer	GTCTCCTCTGACTTCAACAGCG
		Reverse Primer	ACCACCCTGTTGCTGTAGCCAA

Table S3. Anti-viral efficacy of herbs in Vero 9013

Virus	Viral titer (log 10 PFU/mL								
	Control	HD				AC			
		10 mg/mL	5 mg/mL	1 mg/mL	0.1 mg/mL	10 mg/mL	5 mg/mL	1 mg/mL	0.1 mg/mL
JEV	7.33 ± 0.09	0	5.80 ± 0.16	6.95 ± 0.15	7.03 ± 0.16	0	5.33 ± 0.16	7.03 ± 0.12	7.06 ± 0.10
ZIKV	6.51 ± 0.28	0	4.62 ± 0.19	5.92 ± 0.26	5.99 ± 0.22	0	3.08 ± 1.89	5.83 ± 0.46	5.87 ± 0.33
DENV-2	6.42 ± 0.11	0	2.14 ± 1.59	2.76 ± 2.89	2.32 ± 2.43	0	1.09 ± 1.61	2.21 ± 2.36	4.56 ± 2.21

Vero9013 cells were incubated with each virus at MOI 0.01 for 1h, and unbound viruses were removed by washing with EMEM to synchronized the replication, then treated with each herb at a range of concentrations (10, 5, 1, and 0.1 mg/mL). Supernatants were collected to determine infectious viral titers at 72phi for SARS-CoV-2, JEV and ZIKV, and 96 h p.i. for DENV-2 respectively.

Viral titers are expressed as mean ± standard deviation log10 PFU/mL. Viral titers were titrated using Vero9013 cells. All experiments were performed using duodecuplicate (12-replicates).

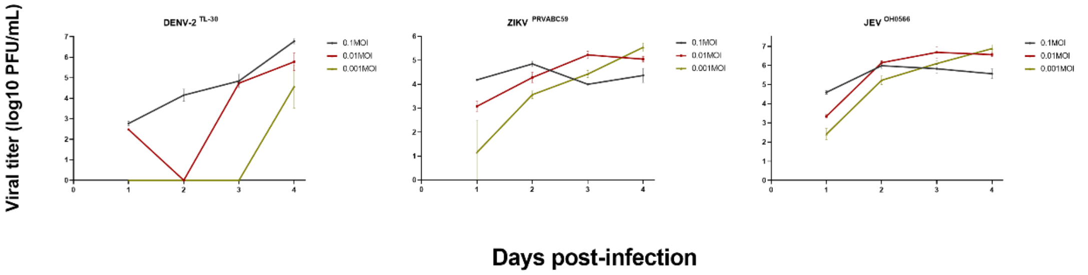

Figure S1. Time-dependent viral titer of Vero 9013 cells inoculated with JEV, ZIKV, and DENV-2 at different virus concentrations. Vero9013 cells were infected with viruses for 1h and unbound viruses was removed by washing with EMEM, and viral titers in supernatant were quantified by plaque assay, performed in duplicate (n=2). Each data point represented the average viral titer ± standard deviation (SD) for the viruses infected vero9013 cells at four time points (Day-1 (24 h p.i.), Day-2 (48 h p.i.), Day-3 (72 h p.i.), and Day-4 (96 h p.i.) post infection). MOI 0.1 (black), 0.01 (red), and 0.001 (yellow).

(A) HD (B) AC

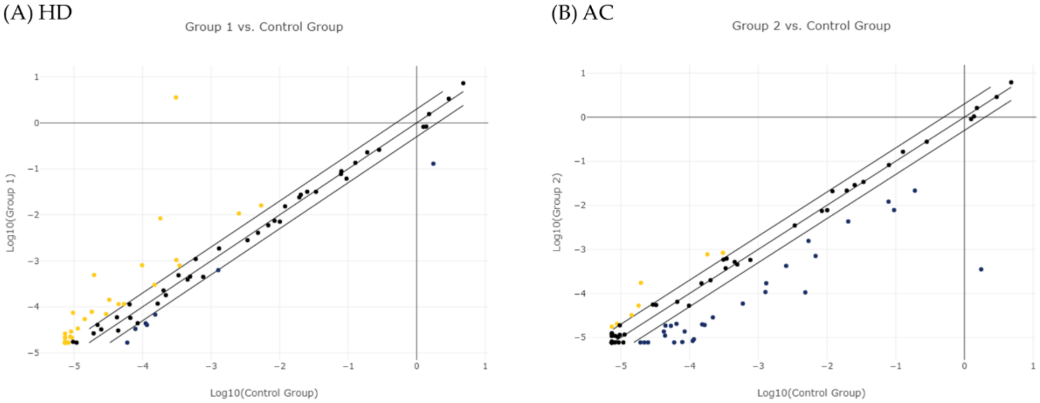

Figure S2. Relative expression comparison of 84 genes involved in human inflammatory cytokines and receptors. (A) Scatterplot analysis depicting the differentially gene expression of inflammatory cytokines and receptors following HD (5mg/mL) treatment against DENV-2 TL-30 (MOI=1) infection in T89G cells at 6-hour post-treatment, compared to untreated viral infection control. (B) Heatmap of gene expression in AC treated samples.

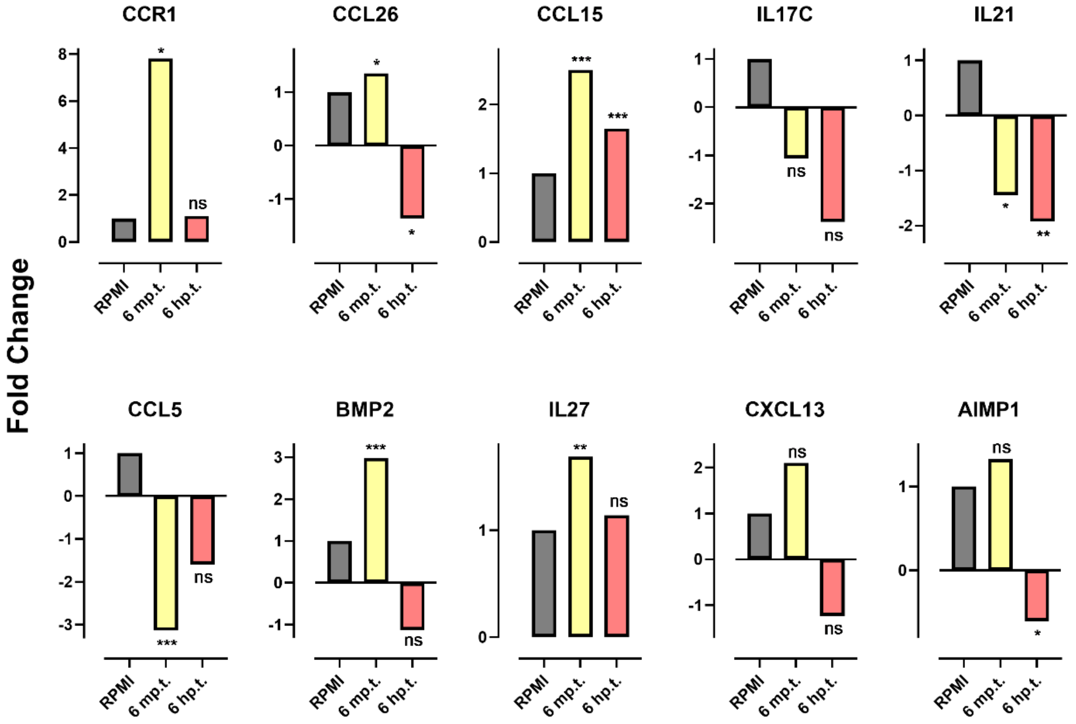

Figure S3. The fold changes of the selected inflammatory genes in DENV-2 TL-30 infected T98G cells relative to T98G cells. T98G cells were incubated with DENV-2 TL-30 at MOI=1 for 1h, and the unbound virus were removed by washing with RPMI, then treated with RPMI supplemented with 15% FCS. Cells were collected to determine gene expression level at 6-minute and 6-hour post-treatment. (A) Selected genes for which treatment with HD. (B) Gene expression in AC treated samples. *$p < 0.05$; ** $p < 0.01$; *** $p < 0.001$; **** $p < 0.0001$.

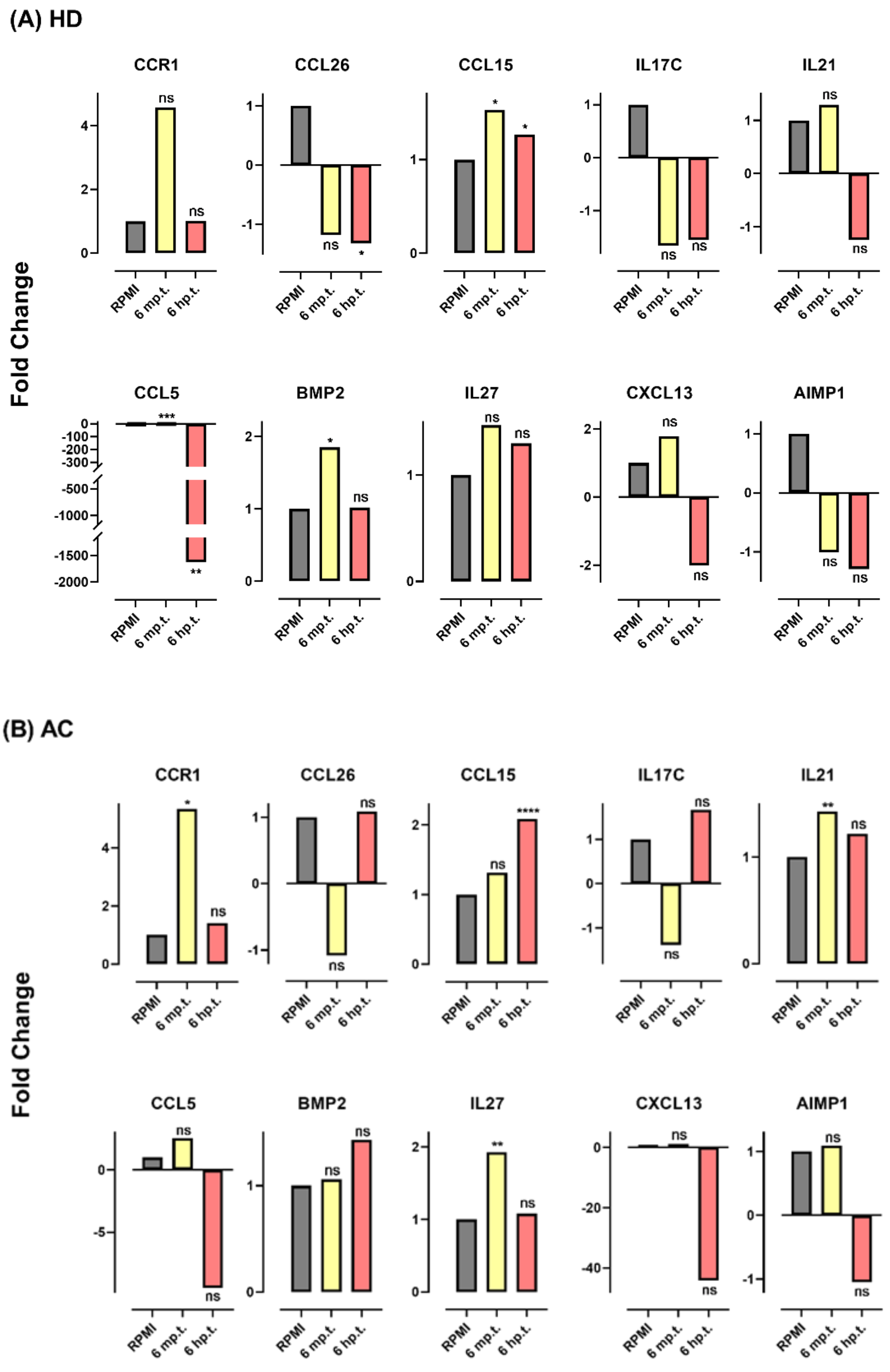

Figure S4. The fold changes of the selected inflammatory genes in herb treated DENV-2 TL-30 infected T98G cells relative to T98G cells. T98G cells were incubated with DENV-2 TL-30 at MOI=1 for 1h, and the unbound virus were removed by washing with RPMI, then treated with each herb at a concentration of 5 mg/mL or RPMI supplemented with 15% FCS. Cells were collected to determine gene expression level at 6-minute and 6-hour post-treatment. (A) Selected genes for which treatment with HD. (B) Gene expression in AC treated samples. *$p < 0.05$; ** $p < 0.01$; *** $p < 0.001$; **** $p < 0.0001$.

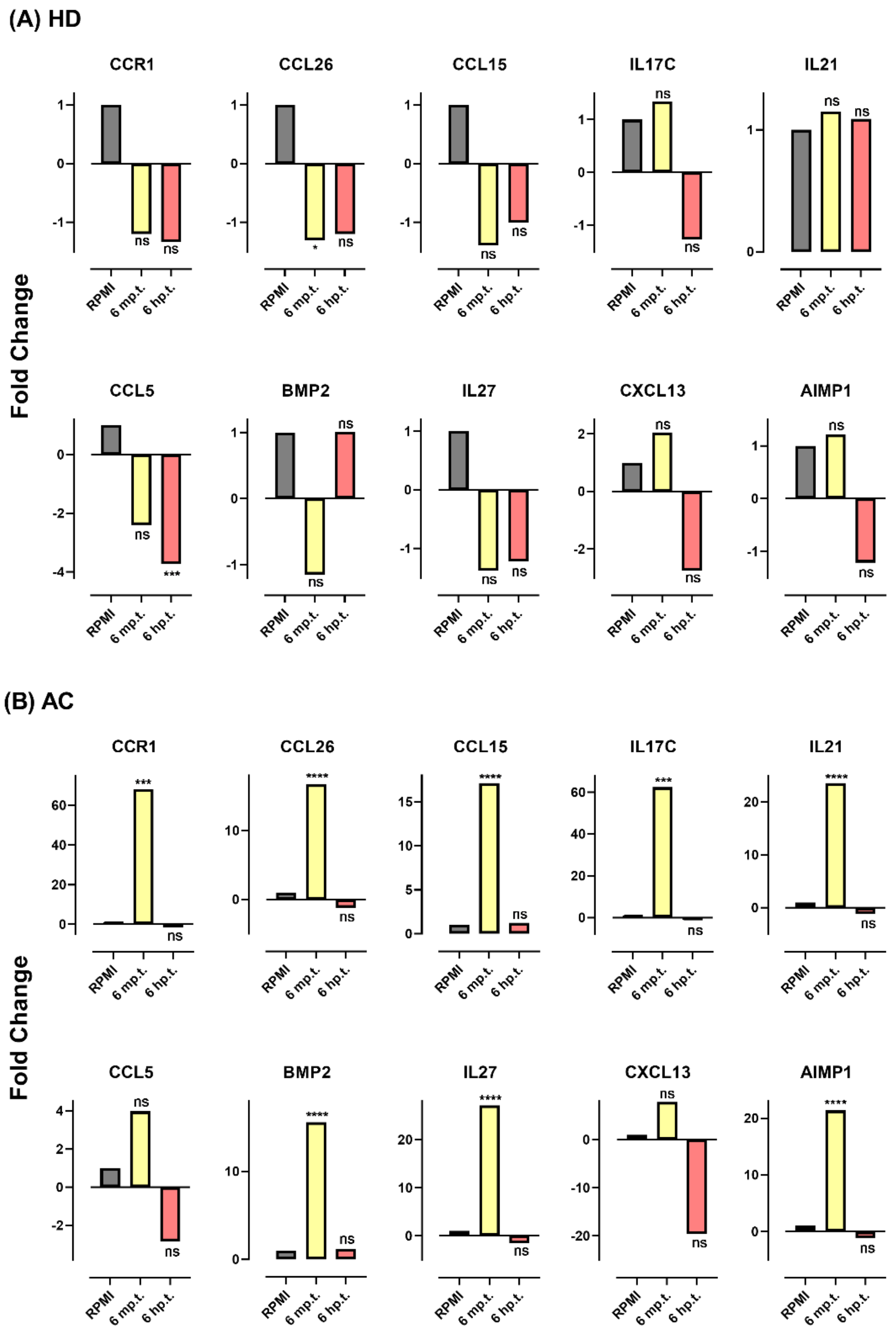

Figure S5. The fold changes of the selected inflammatory genes in herb treated T98G cells relative to T98G cells. T98G cells were treated with each herb at a concentration of 5 mg/mL or RPMI supplemented with 15% FCS. Cells were collected to determine gene expression level at 6-minute and 6-hour post-treatment. (A) Selected genes for which treatment with HD. (B) Gene expression in AC treated samples. *$p < 0.05$; ** $p < 0.01$; *** $p < 0.001$; **** $p < 0.0001$.

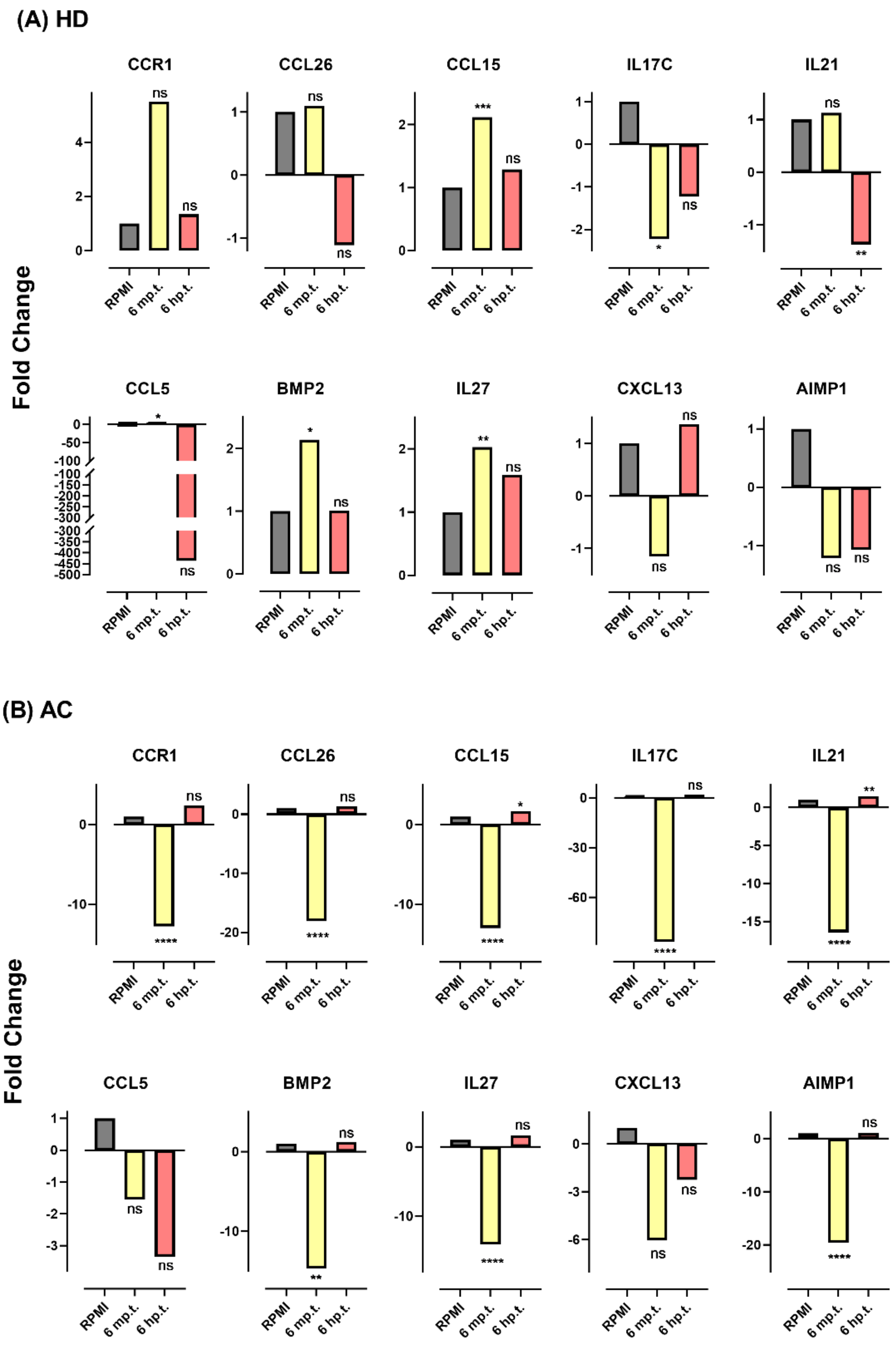

Figure S6. The fold changes of the selected inflammatory genes in herb treated DENV-2 TL-30 infected T98G cells relative to herb treated uninfected cells. T98G cells were incubated with or without DENV-2 TL-30 at MOI=1 for 1h, and washed with RMPI, then treated with each herb at a concentration of 5 mg/mL. Cells were collected to determine gene expression level at 6-minute and 6-hour post-treatment. (A) Selected genes for which treatment with HD. (B) Gene expression in AC treated samples. *p < 0.05; ** p < 0.01; *** p < 0.001; **** p < 0.0001.
